# HSV-2 Infection Enhances Zika Virus Infection of Primary Genital Epithelial Cells Independently of the Known Zika Virus Receptor AXL

**DOI:** 10.3389/fmicb.2021.825049

**Published:** 2022-01-20

**Authors:** Germán G. Gornalusse, Mengying Zhang, Ruofan Wang, Emery Rwigamba, Anna C. Kirby, Michael Fialkow, Elizabeth Nance, Florian Hladik, Lucia Vojtech

**Affiliations:** ^1^Department of Obstetrics & Gynecology, University of Washington, Seattle, WA, United States; ^2^Molecular Engineering and Sciences Institute, University of Washington, Seattle, WA, United States; ^3^Department of Chemical Engineering, University of Washington, Seattle, WA, United States; ^4^Vaccine and Infectious Disease Division, Fred Hutchinson Cancer Research Center, Seattle, WA, United States; ^5^Division of Allergy and Infectious Diseases, Department of Medicine, University of Washington, Seattle, WA, United States

**Keywords:** Zika, herpes, AXL, co-infection, STI, genital

## Abstract

Zika virus (ZIKV) is transmitted to people by bite of an infected mosquito and by sexual contact. ZIKV infects primary genital epithelial cells, the same cells targeted by herpes simplex virus 2 (HSV-2). HSV-2 seroprevalence is high in areas where ZIKV is endemic, but it is unknown whether HSV-2 increases the risk for ZIKV infection. Here, we found that pre-infecting female genital tract epithelial cells with HSV-2 leads to enhanced binding of ZIKV virions. This effect did not require active replication by HSV-2, implying that the effect results from the immune response to HSV-2 exposure or to viral genes expressed early in the HSV-2 lifecycle. Treating cells with toll-like receptor-3 ligand poly-I:C also lead to enhanced binding by ZIKV, which was inhibited by the JAK-STAT pathway inhibitor ruxolitinib. Blocking or knocking down the well-studied ZIKV receptor AXL did not prevent binding of ZIKV to epithelial cells, nor prevent enhanced binding in the presence of HSV-2 infection. Blocking the α5 integrin receptor did not prevent ZIKV binding to cells either. Overall, our results indicate that ZIKV binding to genital epithelial cells is not mediated entirely by a canonical receptor, but likely occurs through redundant pathways that may involve lectin receptors and glycosaminoglycans. Our studies may pave the way to new interventions that interrupt the synergism between herpes and Zika viruses.

## Introduction

The presence of any sexually transmitted infection (STI) itself indicates risk for other STIs (Menezes et al., 2018). It makes sense that a behavioral risk factor (unprotected sexual activity) increases risk for all STIs. However, there are also well documented biological pathways whereby infection with one pathogen increases susceptibility for another (Ward and Rönn, 2010; Kalichman et al., 2011). Herpes Simplex Virus 2 (HSV-2) is a common STI with global prevalence estimated at 11.3% (Looker et al., 2015). The association between HSV-2 infection and HIV acquisition risk is particularly well-studied, with meta-analysis indicating a two–fivefold increased risk of HIV in individuals with prevalent or incident HSV-2 infection. HSV-2 clearly has a profound impact on the genital tract, which may influence risk of infection with other STIs.

Epithelial cells of the genital tract are the initial targets of sexually transmitted HSV-2 and are frequently re-infected following release of virus from latently infected nerve cells. Reactivation leads to replication in epithelial cells, death of the infected epithelial cells and subsequent shedding of viral particles, and an immune response to viral replication. In some individuals, symptomatic HSV-2 lesions occur during bouts of reactivation, but most episodes of reactivation and shedding go unnoticed (Wald et al., 1995; Cunningham et al., 2006; Schiffer and Corey, 2013). Such asymptomatic shedding can be very frequent; studies suggest HSV-2 shedding on 2–25% of days (Wald et al., 1995; Mark et al., 2008). Dendritic cells and memory HSV-2 specific T cells persist at sites of genital tract infection and are thought to contribute to the rapid containment of infected cells (Schiffer and Corey, 2013). Epithelial cells themselves recognize infection and initiate an antiviral program (Zhou et al., 2015), but the virus also encodes an arsenal of evasive strategies that dramatically alter the host cell transcriptome and proteome (Tognarelli et al., 2019). The overall effect of HSV-2 infection on host susceptibility to other STIs is therefore complex and likely to be different for each STI.

HSV-2 and Zika virus (ZIKV) produce, along with other pathogens referred to as TORCH (toxoplasmosis, rubella virus, cytomegalovirus, HSV), congenital infections with severe fetal abnormalities, including intrauterine growth restriction and microcephaly (Mlakar et al., 2016; Walker et al., 2020). ZIKV outbreaks, although not frequent during recent years, remain a threat for pregnant women. An effective vaccine to prevent ZIKV infection has not been licensed yet. Geographically, HSV-2 and ZIKV endemic areas overlap (Moreira-Soto et al., 2018). In areas where microcephaly due to ZIKV is common 40% of women in childbearing age are seropositive for HSV-2 (Miranda et al., 2014; Teixeira et al., 2016; Lima et al., 2018). Though more often transmitted by the bite of an infected mosquito, sexual transmission of ZIKV, primarily from men to their sexual partners, is well-established. It is estimated that between 3 and 15% of ZIKV infections result from sexual transmission (Gao et al., 2016; Towers et al., 2016; Sasmal et al., 2018). It has been shown that the virus persists in semen for up to 6 months post-infection, and is present in asymptomatic individuals (Atkinson et al., 2017). Furthermore, sexual transmission of ZIKV may result in increased risk to the fetuses of pregnant women, potentially leading to lifelong disabilities caused by *in utero* ZIKV infection (Yockey et al., 2016; Winkler et al., 2017; Duggal et al., 2018). We have reported that ZIKV readily infects primary epithelial cells of the vagina, ectocervix and endocervix (Wang R. et al., 2020), the same cells targeted by HSV-2.

Clinical reports indicate the coinfections of ZIKV and other pathogens are possible (Grayo, 2021). There is scarce data explaining the mechanisms underlying viral synergism; to the best of our knowledge, only one study explored HSV-ZIKV coinfection and the cellular model was first-trimester trophoblastic cells (Aldo et al., 2016), which are not typical host cells for HSV. In these cells derived from placenta, the authors showed that ZIKV is cytotoxic and blocks type I interferon signaling; in contrast, we did not observe cytopathic effects in our model of primary epithelial cells from the female tract (Wang R. et al., 2020), Thus, there is a gap of knowledge in regards to the pathogenesis of ZIKV in genital tissues. Because HSV-2 infection induces cellular changes that could both enhance (e.g., induction of entry receptors, disruption of the monolayer architecture) and inhibit ZIKV infection (e.g., upregulation of immune factors like IL-8 and IL1β), we tested whether acute infection with HSV-2 alters the susceptibility of target cells to ZIKV using an *in vitro* model of ZIKV challenge.

## Materials and Methods

### Generation and Culture of Primary Genital Epithelial Cells

The protocol for obtaining genital tissues from patients was approved by the Institutional Review Board (IRB) of both University of Washington and the Fred Hutchinson Cancer Research Center in Seattle, WA, United States with informed consent signed by each patient. Cultures of primary untransformed epithelial cells were generated as we previously reported (Hladik et al., 2015). Epithelial cells were maintained by culture in the presence of mouse fibroblast feeders and the inhibitor of the Rho-associated protein kinase (ROCK) Y-27632. The culture medium (“F-medium”) consisted of a mixture 3:1 v/v of F12 media and DMEM high glucose containing 5% heat-inactivated fetal bovine serum, 0.4 μg/ml hydrocortisone, 5 μg/ml insulin, 8.4 ng/ml cholera toxin, 10 ng/ml epidermal growth factor, 24 μg/ml adenine, 100 U/ml penicillin/100 μg/ml streptomycin, 2 mM L-glutamine and 3.33 μg/ml (10 μM) Y-27632 (Tocris Bioscience). Cells were grown in a humified incubator at 37°C with 5% CO_2_. To separate feeder cells from epithelial cells, flasks were rinsed with PBS and then incubated at 37°C for 10 min with versene solution (0.48 mM EDTA in PBS, Thermo Fisher Scientific). The keratinocytes, which remained attached to the flask, were passed as single cells after treating the adherent monolayer with 0.25% trypsin/EDTA with phenol red (Thermo Fisher Scientific) for 5 min at 37°C; trypsin was inactivated by dilution with medium containing 10% FBS and placing the cell suspension on ice. After centrifuging cells at 300 g for 5 min, cells were counted. For ZIKV infection experiments cells were plated in the absence of fibroblast feeder cells.

### Virus Preparation and Determination of Viral Titer

All HSV strains were prepared in Dr. David Koelle’s laboratory. HSV-2 strain 186 was originally isolated from a genital lesion from an individual who was attending a sexually transmitted disease clinic in Houston, TX, United States (Rawls et al., 1968). For coinfection experiments, we also included a thymidine kinase-deficient HSV-2 strain 333 (tk–HSV-2) expressing GFP (Kit et al., 1983). The primary isolates were passed on Vero cells (American Tissue Culture Collection) to prepare the viral stocks. Titration of HSV stocks was also done in Vero cells. The titers of the stocks used in this study were 1.02 × 10^9^ and 1.41 × 10^7^ PFU/ml for HSV-2 186 and HSV-2 333 GFP, respectively. UV inactivation was done irradiating an aliquot of HSV-2 (in an open Petri dish) under the UV light of a bio cabinet for 30 min at room temperature.

Zika virus strain VR-1848 was purchased from the American Type Culture Collection (ATCC). Viral stocks were generated by infecting Vero cells (obtained from the laboratory of Dr. David Koelle, University of Washington) at 70% confluence with an approximate multiplicity of infection (MOI) of 0.1 of virus in Dulbecco’s Modified Eagle Media for 1.5 h, then removing inoculum and replacing with VP-SFM serum-free low protein medium (Gibco). Supernatants were harvested when Vero cells had visible cytopathic effects, at 4- or 5-days post infection. Stocks were centrifuged at 350 × *g* for 10 min to pellet cell debris, then concentrated sevenfold in Centricon plus 70–100 kDa cutoff centrifugal filter unit by spinning at 3,400 RPM at 4° for approximately 1 h. Mock infected Vero supernatants were generated and concentrated in the same way. Stocks were aliquoted and stored at −80°. All experiments were done with virus passaged twice in Vero cells. Viral titer was determined as plaque-forming units (PFU) per ml as described below. Fortaleza stocks were obtained from the laboratory of Dr. Michael Gale, Jr. and propagated in the same way.

Five hundred thousand Vero cells were plated per well on six-well plates in D10 media (DMEM, 10% FBS, 2 mM L-glutamine, 50 U/ml penicillin, and streptomycin) 1 day prior to infection. Virus-containing culture media were diluted in DMEM media then added to the Vero cells (1 ml/well). Cells were incubated for 1.5 h then inoculum was removed. D10 media with 0.4% agarose was added to each well (2.5 ml/well). After agarose solidified for 5 min, plates were incubated at 37°C for 3–5 days until the plaques became visible. Plates were fixed with 10% neutral buffered formalin for at least 2 h in room temperature. Agarose was removed after fixation and 0.5% crystal violet in 10% ethanol was added to the wells for staining then washed off with water prior to counting plaques by eye.

### Herpes Simplex Virus and Zika Virus Infections

Genital epithelial cells were seeded on day 1 in F medium supplemented with 10 μM Y-27632 without mouse fibroblast feeder cells; we plated 1 × 10^5^ cells/well in a 12-well plate. Infections with HSV were done on day 3 of culture; the multiply of infection (MOI) used was 0.1. We infected the cells for 1 h at 37°C with a solution containing HSV-2 or an equivalent volume of Vero mock control and consisting of Hanks’ Balanced Salt Solution (HBSS), supplemented with calcium and magnesium (Gibco), 1% V/V heat-inactivated FBS and 0.01% W/V glucose (Sigma-Aldrich). After 1 h of incubation, viral or mock inocula were aspirated, discarded and replaced with 1% heat inactivated FBS-DMEM supplemented with (100 U/ml penicillin/100 μg/ml streptomycin, and 2 mM L-glutamine for D1 medium) with or without 2 μg/ml acyclovir (ACV). The extent of infection was evaluated at 24 h by phase microscopy and by qPCR, as done before (Gornalusse et al., 2020).

On the day of infection, Zika virus (VR-1848 or Fortaleza; Vero mock control), diluted in plain DMEM to 350 μl with an MOI of 1, was added to the cells and incubated for 1.5 h. In some experiments, the incubation was done on ice and in others at 37°C. Inoculum was aspirated and washed away after incubation and replaced by F media with ROCK inhibitor. Cells were lysed in 350 μl of RLT lysis buffer (Qiagen) containing 1% V/V beta-mercaptoethanol for RNA analysis or fixed with 4% paraformaldehyde for 15 min for immunofluorescence staining.

### Viral RNA Quantification With RT-Digital Droplet PCR and Quantitative Real-Time PCR

To quantify ZIKV copy number, we used a two-step reverse transcription droplet digital PCR (RT-ddPCR) approach to detect ZIKV genomes (positive strand). RNA of infected cells was extracted with Qiagen RNeasy Plus Mini kit (Qiagen) according to manufacturer’s instructions and eluted with 30–40 μl RNase-free water. Genomic DNA was eliminated using the gDNA eliminator columns that are included in the kit. RNA concentration was quantified with NanoDrop spectrometer (Thermo Fisher). Between two hundred and one thousand nanograms of extracted RNA were used as template for cDNA synthesis with High-Capacity cDNA Reverse Transcription Kit (Applied Biosystem) and random hexamers. Upon completion of cDNA synthesis, samples were diluted 1:5 by adding 180 μl of molecular biology grade water. Five microliters of 1:5 diluted cDNA was used for ddPCR droplet generation. ddPCR reaction was done in a total volume of 22 μl of a mixture that contained 11 μl of ddPCR Supermix for Probes with no dUTP (Bio-Rad), 1.1 μl of 20 × 6-carboxyfluorescein (FAM)-labeled target ZIKV quantitative PCR (qPCR) probe/primers, 1.1 μl of 20X HEX-labeled housekeeping RPP30 gene-specific Taqman gene expression probe/primers (Integrated DNA Technologies) and 5 μl of diluted cDNA. The primers and probe to detect ZIKV RNA were as follows: (Forward primer: 5′-CCG CTG CCC AAC ACA AG-3′, reverse primer: 5′CCA CTA ACG TTC TTT TGC AGA CAT-3′), (Probe: 5′−/56-FAM/AGC CTA CCT/ZEM/TGA CAA GCA GTC AGA CAC TCA A/−3′), and housekeeper gene RPP30 (IDT DNA technologies assay ID Hs.PT.58.19785851). Each assembled ddPCR reaction mixture was loaded in duplicate into the sample wells of an eight-channel disposable droplet generator cartridge (Bio-Rad) and droplet generation oil (Bio-Rad) was added. After droplet generation, the samples were amplified to the endpoint in 96-well PCR plates on a conventional thermal cycler (C1000, Bio-Rad) using the following conditions: denaturation/enzyme activation for 10 min at 95°C, 40–60 cycles of 30 s denaturation at 94°C and 60 s annealing/amplification at 60°C, followed by a final 10 min incubation step at 98°C. After PCR, the droplets were read on the QX200 Droplet Reader (Bio-Rad). Analysis of the ddPCR data was performed with QuantaSoft analysis software version 1.3.1.0 (Bio-Rad). A non-template control well containing ddPCR reaction mix but no cDNA was included to adjust the reaction threshold. Droplets positive for viral RNA were normalized to housekeeper gene copy number.

To correlate ZIKV copy numbers (RT-ddPCR) to threshold cycle values (“Ct”, qPCR), we ran 5 μl of serial dilutions of one of cDNA samples (dilutions 1:4; 1:40; 1:400 and 1:4000) using a qPCR system and the same primers and probes for ZIKV and RPP30 as described before. The master mix contained the following: 11.0 μl of 2 × Absolute Blue qPCR carboxy-X-rhodamine mix (Thermo Fisher Scientific), 1.1 μl of 20 × 6-carboxyfluorescein (FAM)-labeled target ZIKV quantitative PCR (qPCR) probe/primers, 1.1 μl of 20× HEX-labeled housekeeping RPP30 gene-specific Taqman gene expression probe/primers (Integrated DNA Technologies) and 5 μl of diluted cDNA. We ran two technical replicates for both qPCR and RT-ddPCR. Cycling conditions were as follows: initial denaturation and activation of Taq DNA polymerase at 95°C for 15 min and 40 cycles of 15 s of denaturation at 95°C and 60 s of annealing/extension at 60°C. Thermocycling was done on a QuantStudio 5 (Applied Biosystems).

### Quantum Dot Labeling of Zika Virus Particles and Visualization of Herpes Simplex Virus-2/QD-Zika Virus Coinfections

The conjugation of quantum dots (QD) with ZIKV particles was done via a slightly modified version of a previously published protocol through hydrazine–aldehyde-based click chemistry (Zhang et al., 2020). Briefly, 8 μM QDs functionalized with PEG-NH_2_ (QD-PEG-NH_2_) (Thermo Fisher) was quickly mixed with 0.5 mg of Sulfo-S-4FB (TriLink BioTechnologies) in 1 × PBS to make a 500 μL mixture and incubated for 2 h at room temperature to modify 4FB ligand on QDs (QD-4FB). 500 μL of ZIKV was quickly mixed with 0.5 mg of Sulfo-S-HyNic (TriLink BioTechnologies) in 1 × PBS to make a 1,000 μL mixture and incubated for 2 h at room temperature to modify HyNic ligand on EVs (sEV-HyNic). After incubation, QD-4FB was purified by NAP-5 column (GE Healthcare) following the manufacturer’s instruction. ZIKV-HyNic was purified by Amicon 50 kDa centrifugal filter unit (Millipore Sigma) at maximum speed (2,558 × *g*) for 10 min. Purified QD-4FB and ZIKV-HyNic was mixed and reacted for 2 h at room temperature. After incubation, QD-ZIKV was separated from unconjugated QD-4FB by qEV size exclusion chromatography (SEC) column (iZon). As a negative control, QD-Mock was made by the same procedure as above with ZIKV stock media (Vero cell culture media) substituting ZIKV.

On day 1, 1 million epithelial cells were plated in F-media with 10 μM ROCK inhibitor onto poly-D-Lysine 35 mm TC-treated culture dishes (Corning, Life Sciences). On day 2, cells were infected with HSV-2 333 GFP strain (MOI 0.1) or mock-infected, as explained above. After 1 h incubation, inoculum was replaced with D1 medium and cells were incubated overnight. On day 3, cells were infected with QD-ZIKV (or treated with QD-Mock) for 1.5 h at 37°C; inoculum was removed and dishes were washed with PBS and fixed with 4% paraformaldehyde 2 mM EDTA in PBS. Dishes were incubated in the dark for 15 min, and washed with PBS. Nuclear counterstain was done with 1 ml/dish of a solution 1:1,000 in PBS of TO-PRO™-3 Iodide (Thermo Fisher Scientific). Dishes were stained for 20 min in the dark at room temperature and rinsed once with PBS. Images were acquired using a Nikon A1R confocal microscope (Nikon Inc.).

We took 10 images per slide, for each of 3 cell lines independently infected with HSV-2 and exposed to QD-ZIKV. Total cell area by TOPRO-3 signal, GFP area, and number of QDs identified either overlapping with GFP or not were quantified with Image J software.

### Experiments With Drugs and Antibodies

High Molecular Weight (HMW) Poly(I:C) was purchased from InvivoGen (catalog: tlrl-pic). A stock solution at 1 mg/ml was made using endotoxin free water. Ruxolitinib was obtained from Invivogen (catalog: tlrl-rux); a 20 mM stock solution was made with DMSO. Recombinant human IFN- λ1 (catalog: 300-02L, stock at 8 μg/ml), λ2 (catalog: 300-02K; stock at 8 μg/ml) and β (catalog: 3009-02BC; stock at 5 μg/ml) were from Peprotech. Cilengitide was from Adooq Bioscience; a 5 mM stock was made in DMSO. Mannan was from Sigma-Aldrich (catalog: M7504); a 100 mg/ml stock was made with PBS. Neutralizing antibodies: anti-AXL (R&D, catalog: AF154 RRID: AB_354852), anti-Tyro3 (R&D, catalog MAB859 RRID: AB_2210975), anti MerTK (R&D, catalog AF891 RRID: AB_355691). All drugs and antibodies were used at the concentrations and durations stated in the figure legends.

### AXL siRNA Knock-Down Experiments

On day 1, epithelial cells were plated in a 12-well plate (10^5^ cells/well in 1 ml of F-medium with 10 μM ROCK inhibitor). To perform the knock down, we used the TriFECTa RNAi kit (Integrated DNA Technologies), which included 3 predesigned DsiRNAs (anti-AXL) and a scramble negative DsiRNA control. We made 10 μM stocks of each siRNA using the provided nuclease free duplex buffer. On day 2, we replenished the cells with 1 ml/well of fresh F-medium. We diluted Lipofectamine™ 3000 (Life Technologies) with Opti-MEM Medium (3 or 6 μl of Lipofectamine in 100 μl of medium/well). We prepared a master mix in Opti-MEM containing either the 3 anti-AXL siRNAs or the scrambled negative control (also 100 μl of medium/well). We added the diluted siRNA master mix to the diluted Lipofectamine (1:1 ratio), vortexed and incubated 15 min at room temperature. We added 200 μl of each siRNA-lipid complex to the wells containing 1 ml of F-medium. The final concentration we used was 1 or 10 nM of each anti-AXL siRNA and 10 nM for the scrambled negative control. In some experiments, we added only diluted Lipofectamine to examine toxicity. To test the efficiency of knock down, some wells were incubated for 24 h and on day 3, RNA lysates were prepared to determine AXL mRNA levels by qPCR. On day 4, we did HSV-2 333 GFP infections as described above. On day 5, we conducted ZIKV binding experiments of HSV-2 pre-infected cells and we also obtain protein lysates for analysis of AXL by Western blotting, as described below.

### Western Blotting

Whole cell protein extracts were obtained by treating cellular pellets with RIPA buffer containing protease inhibitor (Santa Cruz catalog sc-24948) on ice for 30 min followed by 10 min centrifugation at 12,000 rpm 4°C. Total protein concentrations were determined by Pierce™ BCA assay (Thermo Fisher) following the manufacturer’s instructions. For western blotting, 25 μg of total protein were heated to 95°C for 5 min in 1× DTT-containing sodium dodecyl sulfate (SDS) sample buffer (NP0007, Thermo Fisher) and electrophoresed at 200 V for 25 min in Bolt™ 4–12% NuPage Bis-Tri-polyacrylamide gel (NW04210, Thermo Fisher) followed by transfer to Immobilon polyvinylidene difluoride membranes (LC2002, Thermo Fisher) and blocking for 1 h with 5% blotting-grade blocker (Bio-Rad). A pre-stained protein ladder was included alongside the samples for 10–180 kDa MW reference (PageRuler™, Thermo Fisher). The primary monoclonal antibodies used for immunoblotting were: anti-AXL (santa cruz sc-166269 RRID: AB_2243305, used at 1:100) and anti-Actin (sc-47778, RRID: AB_2714189, used at 1:200). Secondary goat anti-Mouse IgG-HRP (Thermo Fisher 31430) was used at 1:10,000. Blots were developed using the SuperSignal™ West Femto developing kit (Catalog: 34095, Thermo Fisher). Chemiluminescence was acquired on a ChemiDoc MP imager (Bio-Rad). We also tested for anti-MER (catalog: AF891, at 1 μg/ml) and secondary anti goat IgG HRP (R&D, HAF109, at 1:1000).

### Zika Virus Co-receptor Quantification by Quantitative Real-Time PCR

The following 20× *Taqman* assays were purchased from IDT:

**Table:** 

Gene	Assay
AXL	Hs.PT.56a.1942285
MERTK	Hs.PT.58.2640315
TYRO3	Hs.PT.58.38778546
Gas6	Hs.PT.58.21535693
RPP30 (housekeeper)	Hs.PT.58.19785851
	

The master mix contained the following: 10.0 μl of 2 × Absolute Blue qPCR carboxy-X-rhodamine mix (Thermo Fisher Scientific), 1.0 μl of 20 × 6-carboxyfluorescein (FAM)-labeled target co-receptor quantitative PCR (qPCR) probe/primers, 1.0 μl of 20× HEX-labeled housekeeping RPP30 gene-specific Taqman gene expression probe/primers (Integrated DNA Technologies) and 5 μl of 1:5 diluted cDNA. We ran two technical replicates for both qPCR and RT-ddPCR. Cycling conditions were as follows: initial denaturation and activation of Taq DNA polymerase at 95°C for 15 min and 40 cycles of 15 s of denaturation at 95°C and 60 s of annealing/extension at 60°C.

### Statistical Analysis

Statistical analysis was done using the GraphPad Prism suite (Prism 9 software for Windows, version 9.1.0).

## Results

### HSV-2 Infection of Primary Genital Epithelial Cells Leads to Increased Zika Virus Binding

**FIGURE 1:**
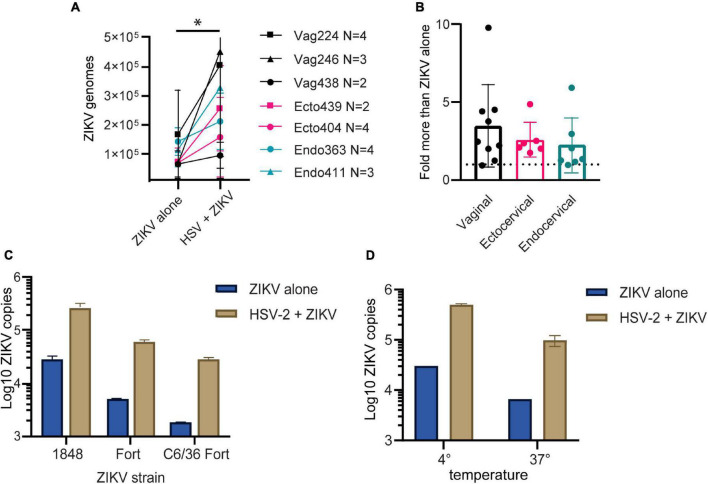
HSV-2 infection leads to enhanced binding of ZIKV virus to cells. **(A)** Cells pre-infected with HSV-2 (strain 186) bind more ZIKV. Three vaginal (in black) and two each ectocervical (in teal) and endocervical (in pink) cell lines were infected with HSV-2 at an MOI of 0.1 for 24 h, or left uninfected. Then cells were exposed to ZIKV at an MOI of 1.0 for 1.5 h, washed 1x with PBS, and lysed for quantification of ZIKV binding and uptake by quantitative RT-PCR. Data are normalized to *RPP30* gene expression and are shown as calculated total genome copies of ZIKV (see section “Materials and Methods”). Each color represents a different tissue origin, and each symbol is a different donor. Results are averaged from 2 to 4 independent experiments for each cell line, error bars are 1 standard deviation. Higher ZIKV in HSV-2 infected wells is significant (*) by Wilcoxan matched-pairs signed rank test, *p* = 0.0156. **(B)** Data from A, plotted as fold more ZIKV in HSV-2 infected wells, for each independent experiment. **(C)** ZIKV binding to HSV-2 infected cells is enhanced with different ZIKV isolates and different producer cells. ZIKV strains 1848 and Fortaleza (Fort) were produced in Vero cells, and Fort was also produced in C6/36 insect cells. Ectocervical cells were infected with HSV-2 GFP strain 333 as described above, then exposed to ZIKV at an MOI of 1 for 1.5 h prior to quantification of ZIKV genomes by RT-qPCR. **(D)** Enhanced binding of ZIKV to HSV-2 infected cells occurs at both 4° and 37°Celsius. Vaginal cells were infected with HSV-2, then exposed to ZIKV for 1.5 h at either 4° or 37° prior to washing and genome quantification by RT-qPCR.

We previously established primary, oncogene-untransformed epithelial cell lines from benign tissues of the vagina, ectocervix, and endocervix, and demonstrated that they can be productively infected by both ZIKV and HSV-2 (Gornalusse et al., 2020; Wang R. et al., 2020). To investigate whether infection with HSV-2 impacts ZIKV replication, we infected epithelial cell lines with HSV-2 at an MOI of 0.1 for 24 h prior to adding ZIKV virions at an MOI of 1.0 for 1.5 h. Cells were washed, then genome copies of ZIKV bound to or taken up by cells was measured with quantitative RT-PCR. We measured binding of ZIKV to cells as a proxy for infection, as HSV-2 causes cytotoxicity at timepoints later than 48 h post-infection and we have previously established a strong correlation between ZIKV binding and infection measured 3 days later (Wang R. et al., 2020). For each experiment we calculated the exact ZIKV copy numbers using a calibration curve done with digital droplet PCR (Supplementary Figure 1A). We tested at least 2 independent cell lines from the vagina, ectocervix, and endocervix, with each donor tested between 2 and 4 times. Results indicate the average ZIKV genome copies per cell line. We observed significantly more ZIKV in cells infected with HSV-2 (*p* = 0.0156) (Figure 1A). We observed this enhancement in binding at 24 h but not at 4 h post HSV-2 infection (Supplementary Figure 1B). Plotted as fold more ZIKV, across all experiments we observed an average of 2.85 more ZIKV genomes in HSV-2 infected cells at 24 h, with no evidence of a difference according to the anatomical origin of the cells (Figure 1B).

To determine if this effect varied by ZIKV strain, we tested 2 different ZIKV isolates, as well as one isolate produced in either Vero or C6/36 mosquito cells, which may have different glycosylation patterns (Routhu et al., 2019). We saw that HSV-2 infection enhanced ZIKV binding for all tested ZIKV strains (Figure 1C), and this effect was independent of normalization to housekeeping gene expression (Supplementary Figure 1C).

With ZIKV added to cells for 1.5 h before analysis, our genome copy assay could be detecting virions both bound to and already taken up into the cell. To determine if prior infection with HSV-2 enhanced specifically ZIKV binding to the cells, we added ZIKV to the cells at either 4°C, which allows virions to bind but not enter the cell, or 37°C, which allows both binding and entry. The temperature was maintained the entire 1.5-h incubation period. We found that with incubation at 37°C we detected less ZIKV genomes than at 4°C, possibly due to genome degradation by the cell upon virus entry. Nonetheless, HSV-2 infection resulted in enhanced ZIKV detection in cells no matter whether incubated at 4 or 37°C (Figure 1D). This suggests that enhanced binding of ZIKV to HSV-2 infected cells is due to a cell surface receptor, and not due to upregulated entry of virus into cells.

### HSV-2 Enhancement of Zika Virus Binding Does Not Require HSV-2 Replication and Occurs in Both HSV-2 Infected Cells and Adjacent HSV-2 Uninfected Cells

**FIGURE 2:**
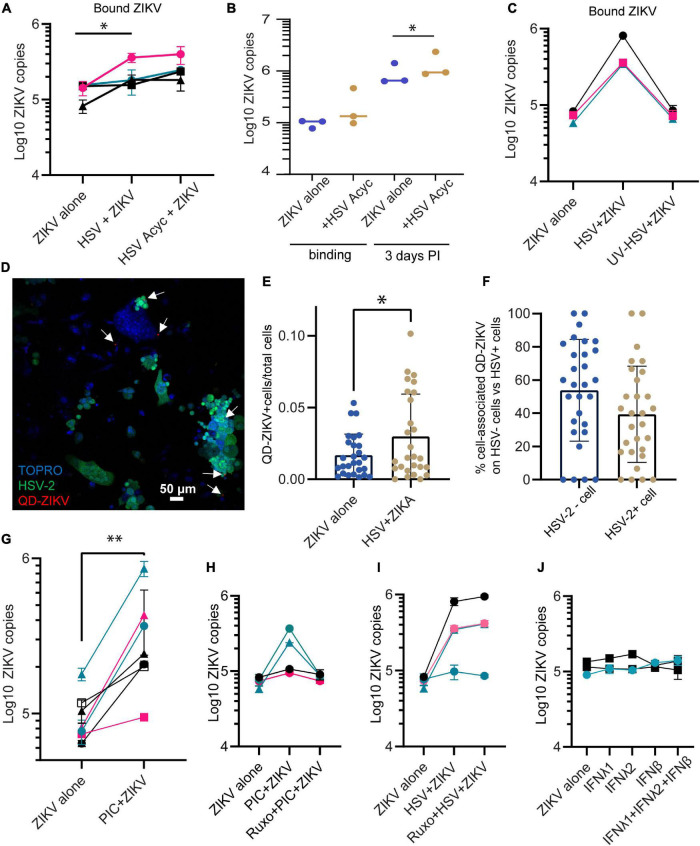
Enhanced binding by ZIKV does not require HSV-2 replication. **(A)** Adding acyclovir at the time of HSV-2 infection still leads to enhanced ZIKV binding. Epithelial cells were mock-treated or pre-infected with HSV-2 strain 186 (MOI 0.1), in the presence or absence of ACV. ZIKV binding was evaluated 24 h post HSV-2 infection. In all plots, each symbol is a different cell line, and colors indicates tissue-of-origin: vaginal in black, ectocervical in teal, and endocervical in pink. *P*-value was calculated using paired *t*-test, *p* = 0.0164, *N* = 4 and is indicated by *. **(B)** HSV-2+ acyclovir results in enhanced ZIKV replication. Epithelial cells were infected as in **(A)**, and lysed immediately (binding) or cultured for 72 h before lysing cells for ZIKV quantification. * indicates significance, *P* value = 0.0233 by ratio paired *t*-test of viral genome copy numbers, *N* = 3. **(C)** UV-inactivated HSV-2 does not result in enhanced ZIKV binding. HSV-2 was treated with UV light or mock treated for 30 min prior to adding to cells. One day following exposure to UV-HSV, ZIKV was added for 1.5 h, then binding of ZIKV to cells was quantified. *N* = 3. **(D)** HSV-2 infection does not correlate with ZIKV binding on a per cell basis. Cells were infected with GFP-expressing HSV-2 strain 333 for 24 h. Then ZIKV virions chemically conjugated to QDs were added for 1.5 h to allow binding. Coverslip wells were washed 1× with PBS, fixed, counterstained with TOPRO-3, and imaged. 10 frames per well were imaged. A representative image with GFP-expressing HSV-2 infected cells is shown; white arrows highlight QD signals. **(E)** QD-ZIKV is more frequently cell-associated in HSV-2^+^ cultures. Total number of QD-ZIKV per frame were counted and divided by the total TOPRO area as a measure of total number of cells. Number of QD/TOPRO area are plotted for each of 10 frames in 3 independent experiments. More cell-associated QD-ZIKV was detected in HSV-2^+^ cultures (∼2-fold more, *p* = 0.044 by *t*-test, indicated by *). Each dot represents a frame. **(F)** Within HSV-2^+^ cultures, cell-associated QD-ZIKV distributes equally between HSV-2^+^ and HSV-2^–^ cells. 10 frames per well were imaged, in 3 independent cell lines. QD signals were counted in a single-color channel, then overlaid with TOPRO and GFP to identify all QD signals associated with a cell. Among all these cell-associated QDs, the percent specifically associated with HSV-2^–^ GFP^–^ or HSV-2^+^ GFP^+^ cells is plotted. Each dot represents a frame. **(G)** Incubating epithelial cells with 10 μg/ml poly I:C [PIC] for 24 h enhances ZIKV binding. Cells were treated with this TLR3 ligand for 24 h. Then cells were exposed to ZIKV at an MOI of 1.0 for 1.5 h, washed 1× with PBS, and lysed for quantification of ZIKV binding and uptake by RT-qPCR. Results are averaged from 2 to 4 independent experiments for each cell line for a total of 7 different cell lines; error bars are 1 standard deviation. *P*-value of 0.003 was determined by paired *T*-test, significance indicated by ^**^. **(H)** Jak1/Jak2 inhibition abrogates PIC- but not HSV-2-mediated enhancement of ZIKV binding. Similar layout as **(G)**, but in some wells cells were preincubated with 2 μM of the specific inhibitor of the protein kinases JAK 1 and 2, ruxolitinib. ZIKV binding was assessed 24 h after PIC treatment. Each line corresponds to a different epithelial cell line. **(I)** Same as **(H)**, but instead of PIC stimulation, cells were infected with HSV-2 strain 333 as described. ZIKV was added at MOI 1.0 for 1.5 h. **(J)** Addition of recombinant soluble IFNs does not change ZIKV infectivity. Epithelial cells were cultured for 24 h with the following cytokines, prior to ZIKV binding: IFNλ1 (100 ng/ml), IFNλ2 (100 ng/ml), IFNβ (100 ng/ml), and a combination of IFNλ1 + IFNλ2 + IFNβ (100 ng/ml each). Each bar shows average + 1 SD of two technical replicates. Experiment done in 3 independent cell lines. Quantification by RT-qPCR.

To test whether the ZIKV enhancing effect of HSV-2 requires the full HSV life cycle, we conducted experiments in the presence of acyclovir. Acyclovir is a nucleoside analog drug that potently inhibits the DNA replication of herpes simplex viruses, but allows initial infection and expression of immediate early and early viral genes (Elion, 1982). We infected epithelial cells with HSV-2 in the presence or absence of acyclovir, and 24 h later added ZIKV for 1.5 h. We found that cells infected with HSV-2 plus acyclovir still bound more ZIKV than HSV-2 unexposed cells (2.00-fold more ZIKV genomes in HSV-2+ acyclovir treated wells compared to cells alone, *p* = 0.0164) (Figure 2A). Acyclovir treatment did not affect ZIKV binding in the absence of HSV-2 (not shown). Acyclovir treatment of HSV-2 exposed cells allowed us to extend ZIKV infection to 72 h by preventing HSV-2 mediated cytotoxicity. We saw a 42% increase in ZIKV genome copies in cells at 72 h post infection, when cells were previously exposed to HSV-2 and acyclovir (*p* = 0.027) (Figure 2B). This demonstrates that enhanced binding of ZIKV to cells leads to corresponding greater viral loads 3 days later. To test whether exposure to HSV-2 viral antigens alone can enhance ZIKV binding to cells, we exposed cells to UV-inactivated HSV-2, then ZIKV for 1.5 h. In this case, we saw no enhanced ZIKV binding (Figure 2C). Taken together, these data demonstrate that the enhancement of ZIKV binding/infection by HSV-2 does not require a full herpes virus replication cycle, but it does require either the expression of early HSV-2 genes or the cellular innate immune response to infection, or both.

To evaluate whether the ZIKV-enhancing effect could be due to an immune factor that is secreted in response to HSV-2 infection, affecting all cells in the culture akin to a paracrine effect, we labeled ZIKV virions with quantum dots (QD) to track their association with cells. Labeling was conducted based on our previously published protocol (Zhang et al., 2020), with some modifications. QD-conjugation did not impair ZIKV viral replication (Supplementary Figure 2). We used a GFP-expressing HSV-2 strain (strain 333), to identify HSV-2 infected cells. We infected cells with HSV-2 for 24 h, then added QD-labeled ZIKV (QD-ZIKV) for 1.5 h, then washed, fixed, and counterstained nuclei with TOPRO-3. GFP-expressing HSV-2 infected cells were clearly observed, as well as punctate QD signal (representative image Figure 2D). In line with the RNA data shown in Figure 1, the ratio of QD-ZIKV/nucleated TOPRO-3^+^ cell was ∼2-fold higher in HSV-infected cultures as compared to mock-infected controls (Figure 2E). Among the HSV-2 infected cultures, we asked whether QD-ZIKV bound with the same frequency to bystander cells (i.e., non-HSV-2 infected) as compared to GFP^+^ HSV-2 infected cells. The data in Figure 2F show no significant difference in the percent of QD-ZIKV binding between HSV-2 positive and negative cells. This demonstrates that cells indirectly exposed to the consequences of active HSV-2 infection in adjacent cells also bind higher numbers of ZIKV virions, indicating that this effect is likely due to a soluble factor released in response to HSV-2 infection.

### Treatment of Epithelial Cells With TLR3 Agonist Poly I:C Also Augments Zika Virus Binding

HSV-2 engages multiple toll-like receptors (TLR) including TLR2, TLR3, and TLR9, and other innate immune recognition pathways (Ma and He, 2014; Jahanban-Esfahlan et al., 2019). We have reported that genital epithelial cells respond robustly to TLR3 stimulation (Gornalusse et al., 2020). To determine if the enhanced ZIKV-binding effect also occurs in response to engaging TLR3, we treated cells with poly I:C, a TLR-3 agonist. Epithelial cell lines were treated with poly I:C for 24 h, then ZIKV added for 1.5 h. Poly I:C treatment significantly enhanced ZIKV binding to cells (*p* = 0.00786, *n* = 7 experiments) (Figure 2G). This suggests that a viral immune recognition pathway such as that triggered via TLR3 can lead to upregulation of a cell surface receptor or co-receptor that binds ZIKV, or downregulation of an inhibitory receptor. Because antiviral interferons (IFNs) are released upon TLR recognition and IFNs signal through JAK-STAT pathways, we used the selective JAK1 and JAK2 inhibitor ruxolitinib (Heine et al., 2013) to determine if IFN signaling was involved in enhanced ZIKV binding. Interestingly, while we found that ruxolitinib abolished enhanced ZIKV binding following poly I:C treatment (Figure 2H), it did not impair enhanced ZIKV binding in response to HSV-2 infection (Figure 2I). In the absence of HSV-2 infection or PIC stimulation, ruxolitinib did not modify ZIKV binding (data not shown). Moreover, addition of purified IFNs to cells did not enhance ZIKV binding (Figure 2J), though the purified IFNs did potently upregulate the interferon-stimulated genes MX1 and ISG15, demonstrating their activity (Supplementary Figure 3). Together, these results suggest that JAK/STAT signaling is involved in poly I:C enhancement of ZIKV binding, but this does not appear to be mediated by direct interferon activity. In contrast, enhancement of ZIKV binding/infection by HSV-2 involves signaling pathways other than the IFN response via JAK/STAT activation.

### Enhanced Zika Virus Binding Occurs Independently of the Known Zika Virus Receptor AXL, and Is Not Impaired by Blocking α5 Integrin or the Mannose Receptor

**FIGURE 3:**
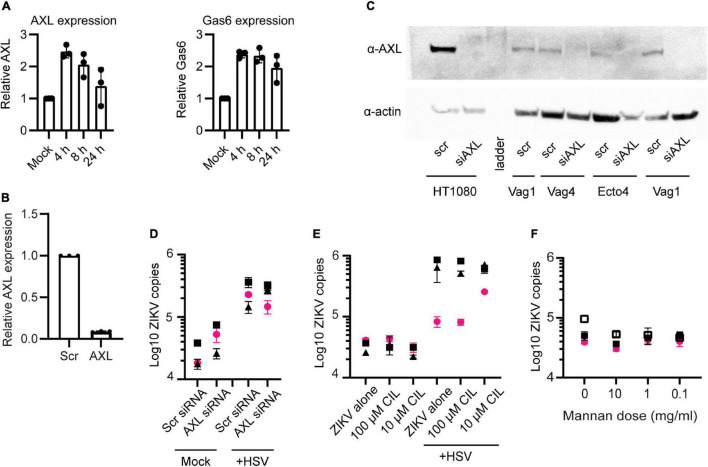
AXL is not essential for ZIKV binding. **(A)** Real-time qPCR analysis of AXL and Gas6 mRNA expression post HSV-2 infection. *Y*-axis shows fold variation relative to mock-infected controls. Gene expression was normalized by RPP30. **(B)** qPCR analysis of AXL mRNA 24 h after knock down by siRNA. Epithelial cells were transfected with a scrambled siRNA (10 nM) or with 3 different siRNAs targeting AXL mRNA (10 nM, each). Lipofectamine 3000 and Trifecta kit (IDT Technologies) was used for the transfection. Data were normalized by RPP30 levels. Data show results from 3 independent experiments done in 3 different cell lines, averaged from 2 technical replicates. **(C)** Western blotting analysis of AXL siRNA knock down. Top: anti-AXL (αAXL; bottom; anti-Actin (αActin). “scr”: scrambled siRNA, used as negative control. “Vag” are vaginal cell lines and “Ecto” is ectocervical. **(D)** ZIKV binding was not affected by AXL knock down. After 72 h of AXL siRNA (or scrambled siRNA) and 24 h post HSV-2 (or mock-treated), cells were exposed to ZIKV for 1.5 h and binding was evaluated by RT-PCR. The figure shows three independent experiments. For **(D–F)**, each symbol is a different cell line and colors indicate tissue of origin: vaginal in black and ectocervical in pink. **(E)** Cilengitide does not block ZIKV binding. Uninfected or HSV-2 infected cells were treated with the RGD binding motif blocking drug cilengitide at indicated concentrations for 1 h at 37°C prior to adding ZIKV for 1.5 h. Binding was evaluated by RT-qPCR, results shown from 3 independent experiments. **(F)** Mannan does not block ZIKV binding to cells. Uninfected cells were incubated with the indicated dose of mannan for 1 h at 37°C prior to adding ZIKV for 1.5 h. Binding was evaluated by RT-qPCR, results shown from 3 independent experiments.

The attachment and entry of flaviviruses to cells is mediated by interactions between the viral E glycoprotein and numerous different cellular surface molecules. It is thought that several cellular proteins are used simultaneously or sequentially to bind to and enter cells (Perera-Lecoin et al., 2014; Agrelli et al., 2019). Among the most well characterized for ZIKV infection is the phosphatidylserine binding TAM family receptor AXL (Meertens et al., 2012; Hamel et al., 2015; Richard et al., 2017; Strange et al., 2019). We wanted to determine if upregulation of AXL or another TAM-family receptor could be responsible for the enhanced ZIKV binding following HSV-2 infection. First, we looked for transcriptional upregulation of AXL and its essential co-factor Growth-arrested-specific 6 (Gas6) using qPCR. We found significant but only modest upregulation of AXL and Gas6 at 4 or 8 h post HSV-2 infection. At 24 h Gas6 expression remained elevated but AXL did not (Figure 3A). Next, we knocked down expression of AXL in our cells using siRNA. Over 95% of AXL expression was blocked at the transcriptional level (Figure 3B), and knockdown of the protein was confirmed by Western blotting (Figure 3C). HT1080 cells, which express AXL, were used as a control for AXL expression and siRNA-mediated knock down. As in other reports, knockdown of AXL reduced ZIKV at 24 h post exposure in HT1080 cells (Supplementary Figure 4A). Surprisingly, and in contrast to reports in some cell types (Richard et al., 2017; Chen et al., 2018; Persaud et al., 2018; Strange et al., 2019), we did not see significant inhibition of ZIKV binding in either HSV-2 infected or uninfected epithelial cells or HSV-2 uninfected HT1080 cells with AXL knockdown (Figure 3D and Supplementary Figure 4A). AXL reportedly can play a dual role in ZIKV infection, serving both to facilitate viral entry and also, via it’s kinase activity, to regulate viral replication in the cell (Strange et al., 2019). We also tested blocking of MerTK and Tyro3, other TAM receptors, with antibodies, and saw no effect on ZIKV binding (Supplementary Figure 4B). For MerTK and Tyro3 this was not surprising, as we did not detect expression of MerTK or Tyro3 in our epithelial cells by Western blot (data not shown). Taken together, this indicates that ZIKV can bind to genital epithelial cells independently of AXL and other TAM receptors, and that AXL upregulation in response to HSV-2 is not responsible for the observed enhanced ZIKV binding.

Other reported attachment factors and receptors for ZIKV include integrins and C-type lectin receptors (CLRs) (Agrelli et al., 2019; Gao et al., 2019). We tested for a role of the α5 integrin in enhanced binding of ZIKV following HSV-2 infection by pre-treating cells with the α5 integrin blocking drug cilengitide (Mas-Moruno et al., 2010). We found no effect on ZIKV attachment to epithelial cells following treatment with this blocker, even after extending the incubation time to 18 h (Figure 3E). To test if CLRs might be involved, we blocked cells with mannan, a polysaccharide recognized by many CLRs (Vendele et al., 2020). Though there was a slight trend toward lower ZIKV binding with the highest dose, the effect was not significant (Figure 3F). Thus, neither α5 integrin or C-type lectin receptors appear to play an essential role in ZIKV binding to genital epithelial cells.

## Discussion

HSV-2 infection is highly prevalent worldwide, with the greatest incidence occurring in women of reproductive age (James et al., 2020). If HSV-2 is acquired during pregnancy, in particular during the third trimester, it can infect the neonate and cause complications including death or long-term disability (Straface et al., 2012). Following the resolution of primary infection, replication of HSV-2 occurs primarily in basal epithelial cells of the mucosa or genital skin following reactivation from latently infected neurons. *In vivo*, sites of HSV-2 reactivation are surrounded by CD4^+^ and CD8^+^ tissue resident T cells and other immune cells which quickly respond to infection and prevent widespread cell death (Zhu et al., 2013). In latently infected individuals, this immune response controls most reactivations of HSV-2 and the direct risk to the neonate is low. However, even well-controlled or treated HSV-2 latent infection is linked to an increased inflammatory state and the concomitant risk for acquisition of other STIs (Gornalusse et al., 2020). HIV is the most studied of these, where increased risk is linked to higher concentrations of HIV target cells at the site of HSV-2 lesions (Zhu et al., 2009); noteworthy, daily acyclovir treatment of HSV-2/HIV co-infected women did not reduce the extent of HIV transmission (Celum et al., 2010). The proinflammatory environment created by HSV-2 reactivation could also impact the infection dynamics of other STIs. Here, we investigated whether active replication of HSV-2 in genital epithelial cells altered pathogenesis of another virus that replicates in epithelial cells, Zika virus.

We found that active HSV-2 infection in epithelial cells of the genital tract led to enhanced binding of ZIKV, and the effect seems to be strain and packing-cell independent, as it was reproduced in two isolates, 1848 and Fortaleza, regardless of packing cell lines. Detection of a higher number of ZIKV genomes happened at the level of binding to cells since it was most apparent following incubation at 4°. A full HSV-2 viral life cycle was not necessary for this effect, as treating cells with acyclovir, which inhibits late gene expression and DNA replication of HSV-2, did not diminish enhanced binding of ZIKV to HSV-2-exposed cells. However, either cell entry or expression of immediate-early HSV-2 genes was required, since UV-treated virus did not enhance ZIKV binding and a short exposure (4 h) of cells to HSV-2 was insufficient to evidence an enhancement in ZIKV binding. Even though the enhancement of *in vitro* binding of ZIKV to target cells was, on average, only 2.5-fold after a single exposure of epithelial cells to HSV-2, it is plausible that *in vivo* the effect can be amplified because of intermittent HSV-2 reactivations with concomitant viral shedding, which happens in the absence of symptoms and even when antiviral drugs are taken (Wald et al., 1997; Johnston and Corey, 2016). Therefore, the repeated inflammatory stimulation of the mucosal epithelium with each HSV reactivation event could increase the susceptibility to ZIKV infection to a higher extent than observable by single-point *in vitro* experiments.

Enhanced binding of ZIKV could occur only in HSV-2 infected cells, or in all cells within the HSV-2 exposed cultures. To distinguish between these possibilities, we needed to identify both HSV-2 and ZIKV infected cells. We used a HSV-2 strain that expresses GFP (HSV-2 GFP 333 UL47) and results in a diffuse cytoplastic GFP signal in HSV-2 infected cells (Koelle et al., 2003). For ZIKV, we utilized a technique we previously developed to label extracellular vesicles (Zhang et al., 2020) to conjugate ZIKV virions with bright and stable quantum dots. Conjugation with QDs did not prevent the ability of ZIKV to replicate in susceptible cells. Using these tools, we confirmed that more ZIKV was detectable in HSV-2-exposed cells, and we observed that ZIKV did not preferentially bind to HSV-2 infected cells. This implied that enhanced ZIKV binding was the result of a soluble factor or factors secreted from HSV-2 infected cells that affected all the cells in the culture. It is technically difficult to take supernatants from HSV-2 infected cultures and separate HSV-2 virions from other soluble components. We attempted two methods, first UV treatment of supernatants, and second, ultracentrifugation of supernatants to preferentially pellet virions. Though both methods significantly depleted HSV-2 plaque-forming units (>95%), addition of the depleted supernatant did not enhance ZIKV binding in new cultures (data not shown). However, UV treatment damages the structure and function of proteins and lipids (Santos et al., 2013). Ultracentrifugation, in addition to pelleting HSV-2 virions, can also deplete proteins including cytokines and pellet extracellular vesicles, which associate with cytokines (Fitzgerald et al., 2018; Benjamin-Davalos et al., 2021). Therefore, a negative result in these assays does not disprove the hypothesis that a soluble factor, or a group of them, secreted from HSV-2 infected cells leads to enhanced binding of ZIKV in neighboring, HSV-2 uninfected cells.

We also tested whether a general anti-viral immune response could mediate enhanced ZIKV binding to cells. We have previously demonstrated that our primary epithelial cells respond robustly to poly I:C, a viral nucleic acid mimic that can engage both TLR3 and RIG-I pathways (Gornalusse et al., 2020). Cells treated with poly I:C also demonstrated significantly enhanced ZIKA binding. Following HSV-2 infection or poly I:C treatment and concomitant production of IFNs, either autocrine or paracrine engagement of IFN receptors leads to a broad antiviral response marked by production of interferon-stimulated genes (Ivashkiv and Donlin, 2014). To determine if IFN signaling played a role in enhanced ZIKV binding to cells, we inhibited the IFN receptor pathway with ruxolitinib (Zhou et al., 2014), a drug that is known to interfere with JAK1/JAK2 tyrosine kinase-mediated signaling pathways. Interestingly, treatment with ruxolitinib abolished enhanced ZIKV binding following poly I:C treatment, but not in response to HSV-2 infection. Treatment of cells with recombinant IFNs did not recapitulate enhanced ZIKV binding, ruling out a sole role for IFN signaling. This is in line with our unpublished results, which demonstrate that *ex vivo* infection of vaginal explants with HSV-2 results in upregulation of not only IFN-λ but also several other immune factors, including CXCL9, CXCL10, IL-1α and TNF-α. Furthermore, a variety of HSV-2 viral proteins engage innate immune pathways in cells, including TLRs, RIG-I-like receptors, the NOD-like receptors, and DNA-dependent sensors (Chew et al., 2009). Thus, while poly I:C and HSV-2 infection both enhance ZIKV binding, the intracellular mechanisms that result in this phenomenon must be distinct.

Since HSV-2 infection enhanced binding of ZIKV to cells, we hypothesized that either a binding receptor or co-factor was upregulated. Flaviviruses are known for being able to use a wide variety of receptors (Agrelli et al., 2019), but the most reported and studied receptor for ZIKV is the TAM family member AXL. It is reported that AXL is required for efficient ZIKV infection in Sertoli cells, brain glial cells, astrocytes, and endothelial cells (Hamel et al., 2015; Liu et al., 2016; Meertens et al., 2017; Richard et al., 2017; Chen et al., 2018; Strange et al., 2019). However, it is dispensable for neural progenitor cells and brain organoids (Wells et al., 2016), and in AXL knockout mice there is no difference in survival rate, viral load, viral distribution, or histological damage to tissues compared to wild-type litter mates following ZIKV infection (Wang et al., 2017). Here, we investigated the role of AXL in ZIKV infection in primary genital epithelial cells. Using siRNA to knock down expression of AXL in our cell lines did not prevent ZIKV binding to cells, either alone or in the presence of HSV-2 infection. However, anti-AXL siRNA was able to inhibit ZIKV in HT1080 cells assessed at 24 h post-infection. Other TAM family receptors include MerTK and Tyro3. Tyro3 is not expressed in our cells (data not shown). MerTK is expressed only minimally, and blocking of MerTK with an antibody did not prevent ZIKV binding.

Our results differ from those reported by Aldo et al. (2016) who showed that the higher sensitivity to ZIKV infection of HSV-2 pre-infected trophoblast cells was due to upregulation of TAM receptors. One potential reason for this discrepancy is that ZIKV may utilize different entry receptors or attachment cofactors for entry in first trimester trophoblast cells versus epithelial cells from the adult female genital tract. Collectively, we demonstrated that AXL is not required for ZIKV infection of vaginal or ectocervical epithelial cells.

The integrin αvβ5 has been shown to mediate ZIKV entry into neural cells (Wang S. et al., 2020; Zhu et al., 2020). Furthermore, herpesviruses bind integrins to promote cell entry (Campadelli-Fiume et al., 2016). To test the role of the α5 integrin, we used cilengitide, a small molecule binding inhibitor of αvβ5 and αvβ3 integrins (Dechantsreiter et al., 1999). We could detect no effect of cilengitide treatment on ZIKV binding. Flaviviruses can also be recognized by mannose receptors such as macrophage mannose receptor (MMR or CD206) (Miller et al., 2008). Because the expression of mannose (and mannose-like) receptors is not confined to macrophages and some dendritic cells (Martinez-Pomares, 2012), but also extends to fibroblasts and keratinocytes (Sheikh et al., 2000; Szolnoky et al., 2001; Fanibunda et al., 2011), we decided to investigate whether this pathway was involved in ZIKV attachment to cells of the genital epithelium. Following incubation with mannan, which is a linear polymer of mannose, we observed a slight but non-significant trend toward less ZIKV binding at only the highest dose tested. This suggests an unidentified lectin could contribute to ZIKV binding, but probably does not play an essential or major role.

Despite testing several possibilities, we were not able to link HSV-2 infection with upregulation of a specific binding receptor. The large number of cell types susceptible to ZIKV infection points to the ability of ZIKV to use numerous host molecules for attachment and entry. In most cells, it is likely that multiple different molecules are used in combination (Agrelli et al., 2019). The interaction between ZIKV and any one host receptor can be weak, but via interaction with multiple receptors avidity reaches the point of successful cellular infection (Agrelli et al., 2019). Therefore, individual receptors are redundant, and could explain why we did not observe a strong exclusive role for any one receptor or attachment factor we tested. Furthermore, HSV-2 infection and the immune response to it profoundly effects normal cellular function, so the enhancement of ZIKV infection following HSV-2 could result from a convergence of different pathways. In future work, it would be interesting to explore the specific attachment and entry receptor combinations and redundancy that allow ZIKV to bind to and enter genital epithelial cells, establishing successful infection. In summary, in this study we have shown that HSV-2 infection of genital epithelial cells resulted in enhanced binding of ZIKV; this effect is independent of any known cofactor(s)/co-receptor(s). Further study of the mechanism of interaction between ZIKV particles and genital target cells could pave the way toward the rational design of new virucidal compounds that block the sexual transmission of Zika virus.

## Data Availability Statement

The raw data supporting the conclusions of this article will be made available by the authors, without undue reservation, to any qualified researcher.

## Author Contributions

LV, GG, RW, and FH: conceptualization. GG, LV, MZ, and EN: methodology. GG, RW, MZ, ER, and LV: investigation. AK and MF: materials and consultation. GG and LV: writing – original draft. GG, LV, RW, MZ, EN, and FH: writing – review and editing. LV and EN: funding acquisition. LV, GG, EN, and FH: supervision. All authors contributed to the article and approved the submitted version.

## Conflict of Interest

The authors declare that the research was conducted in the absence of any commercial or financial relationships that could be construed as a potential conflict of interest.

## Publisher’s Note

All claims expressed in this article are solely those of the authors and do not necessarily represent those of their affiliated organizations, or those of the publisher, the editors and the reviewers. Any product that may be evaluated in this article, or claim that may be made by its manufacturer, is not guaranteed or endorsed by the publisher.
