# Supplementary material for: HSV-2 Infection Enhances Zika Virus Infection of Primary Genital Epithelial Cells Independently of the Known Zika Virus Receptor AXL
Source: Front Microbiol. 2022 Jan 20;12:825049. doi: 10.3389/fmicb.2021.825049 (PMC8811125; doi:10.3389/fmicb.2021.825049)
Supplement: Supplementary file 1 [file Data_Sheet_1.PDF]

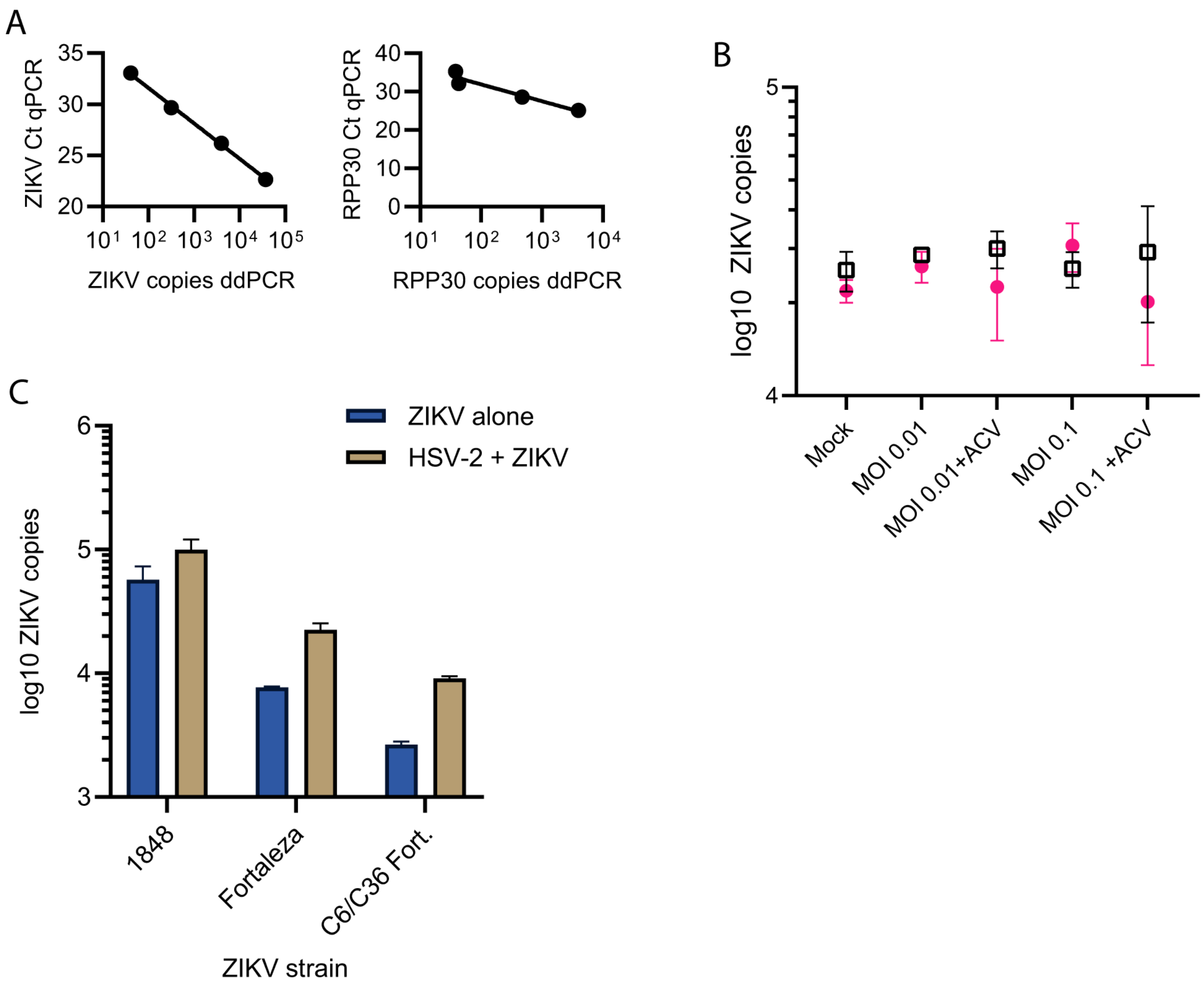

Supplemental Figure 1. (A) Correlation between ZIKV genomes or RPP30 housekeeper copies quantified by digital droplet PCR (x-axis), versus quantitative PCR cycle threshold (y-axis). **(B)** Binding to HSV-2 infected cells is not enhanced 4 hours post HSV-2 infection. Cell were left uninfected or infected with HSV-2 at the indicated MOI (as in Materials and Methods), in the presence or absence of acyclovir (ACV). 4 hours post infection, ZIKV was added at an MOI of 1 for 1.5 hours, then binding assessed by quantitative PCR. Results are from 2 independent experiments. **(C)** ZIKV binding to HSV-2 infected cells is also enhanced when data are not normalized to housekeeper gene expression. Cells were left uninfected or infected with HSV-2 at an MOI of 0.1 for 24 hours. Then ZIKV from 3 different strains was added for 1.5 hours, and binding assessed by quantitative RT-PCR on a set amount of input RNA. Not normalized (absolute) ZIKV genome copies are shown; data from two independent experiments.

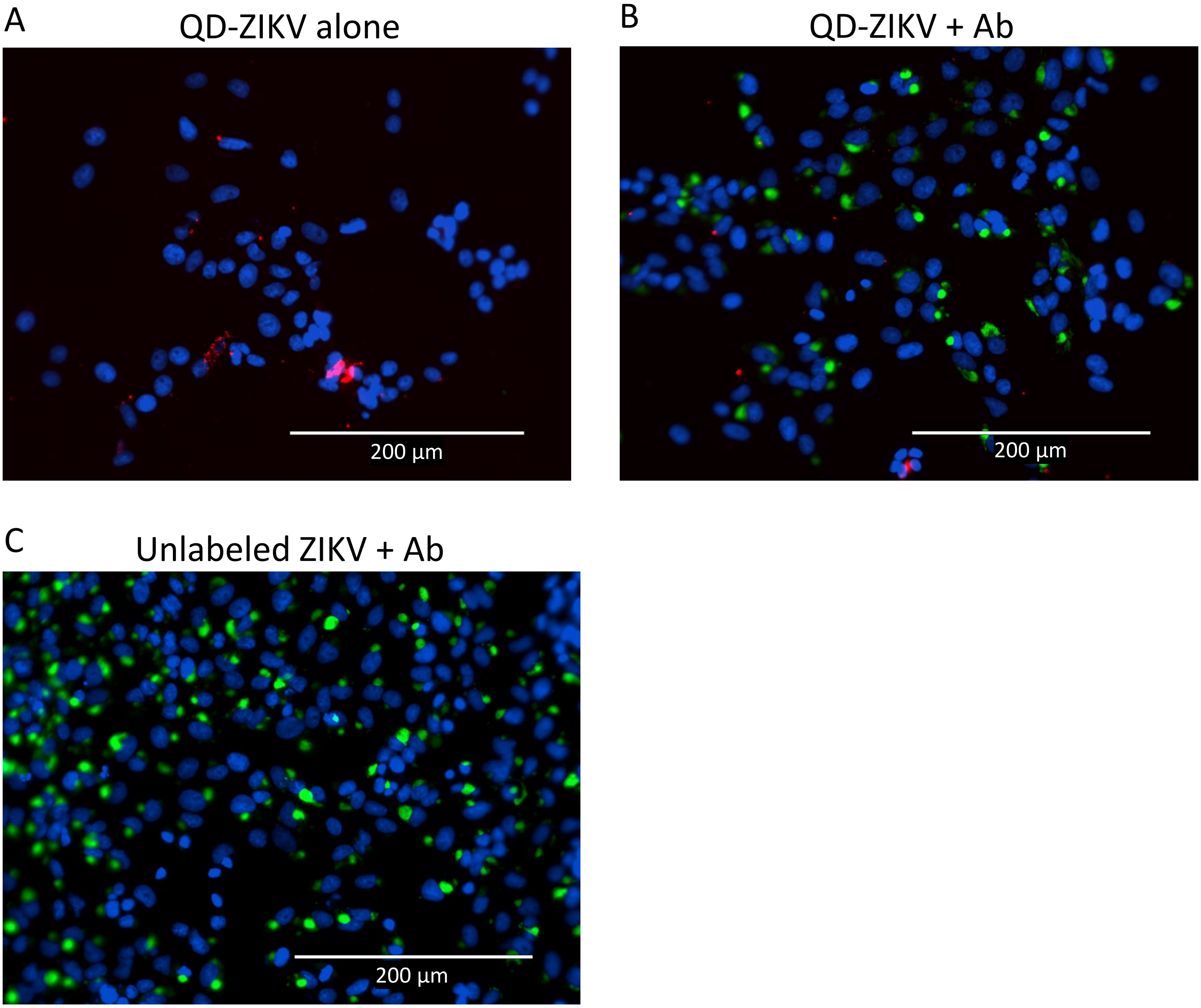

Supplemental Figure 2. QD-tagging does not impair productive infection by ZIKV. ZIKV was conjugated with PEG-quantum dots (QD), as described in the Materials and Methods. Vero cells were infected with QD-tagged ZIKV (panels A & B), or with untagged ZIKV. 3 days later, cells were fixed with 4% PFA, permeabilized, and stained with the 4G2 pan-flavivirus protein E antibody conjugated to Alexa-Fluor 647 (Novus Biologics Antibody D1-4G2-4-15). Nuclei were counterstained with DAPI (in blue). Images were acquired with an EVOS fluorescence microscope, scale bar indicates 200 microns. **(A)** QD-ZIKV alone, red spots are QD signal. **(B)** QD-ZIKV in cells stained with 4G2 antibody. Red spots are QD signal, green staining indicates the presence of ZIKV envelope protein. Expression of ZIKV envelope protein in many cells demonstrates that the QD-conjugation process does not impair productive infection and protein production by ZIKV. **(C)** Untagged ZIKV infection in Vero cells as a control. Green staining indicates the presence of ZIKV envelope protein.

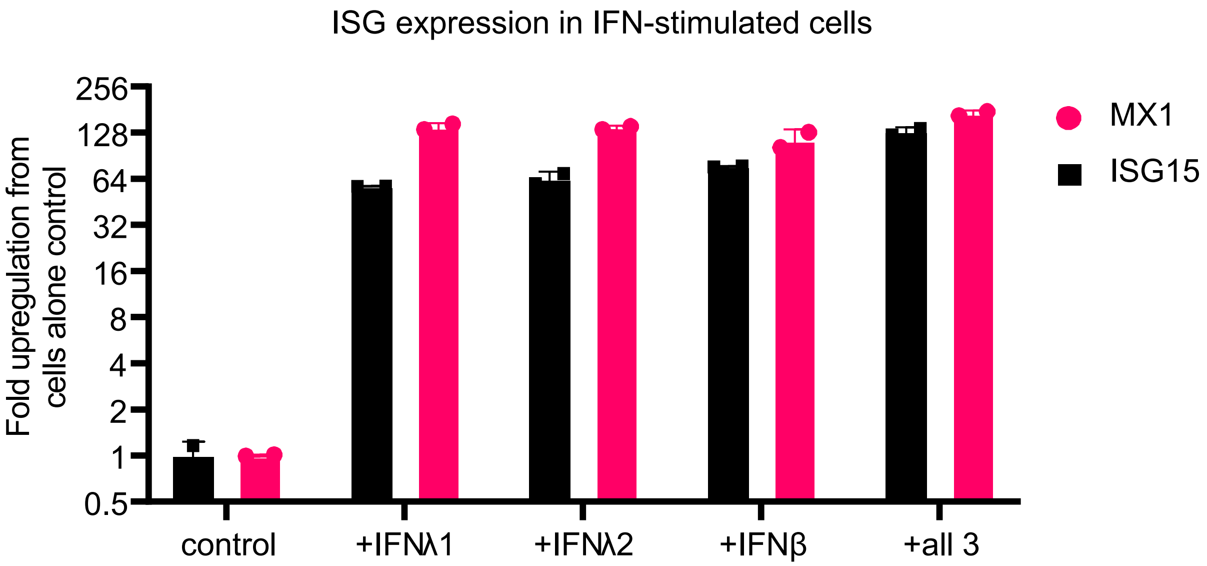

Supplemental Figure 3. Expression of interferon-stimulated genes (ISGs) MX1 and ISG15 in vaginal cells stimulated with recombinant interferons. Cells were stimulated with IFNλ1, IFNλ2, IFNβ at 100 ng/ml each, or all 3 at 100 ng/ml each, for 24 hours. Then cells were lysed, RNA extracted, and expression of MX1 and ISG15 analyzed by RT-qPCR. Results are normalized to the expression of housekeeper gene RPP30 and presented as fold expression relative to unstimulated cells. RT-qPCR done in duplicate, error bars are standard deviation.

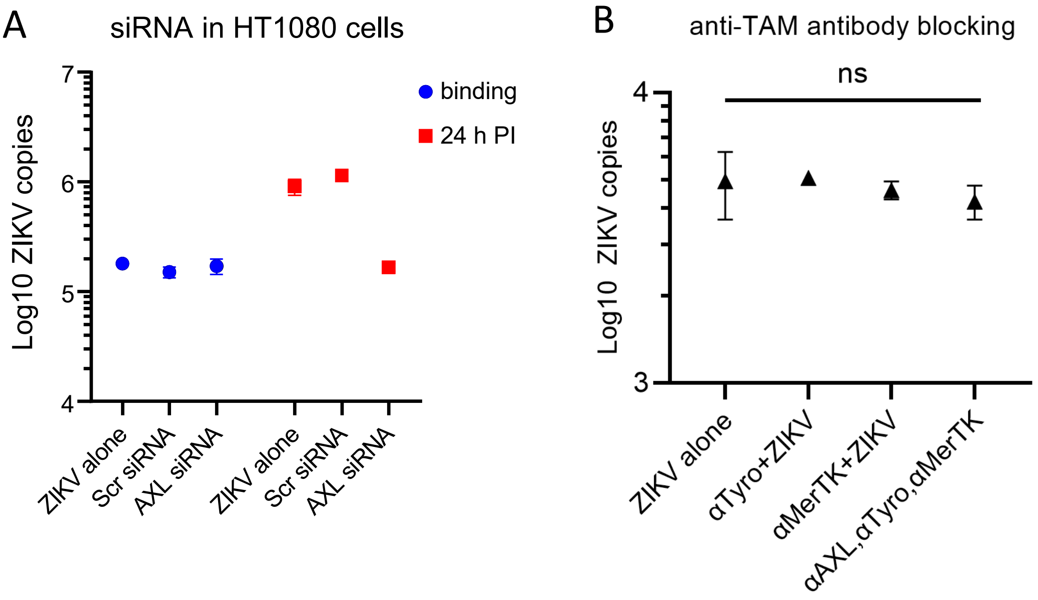

Supplemental Figure 4. siRNA against AXL in HT1080 cells or antibodies against MerTK or Tyro3 on epithelial cells. (A) HT1080 cells were treated with a pool of 3 siRNAs against AXL or with scrambled RNA controls, as described in methods. 48 hours later, ZIKV was added at an MOI of 1 for 1.5 hours, then cells were washed. For the binding timepoint, cells were lysed immediately for RNA extraction. For 24 hours post-infection (PI) fresh media was added, and 24 hours later cells were lysed. ZIKV genomes assessed by qPCR and are presented relative to housekeeper gene RPP30. Conditions done in duplicate. **(B)** Vaginal cells were treated with antibodies against Tyro3 (αTyro) or MerTK (αMerTK), or a combination of αAXL, αTyro, and αMerTK antibodies (all at 10 µg/mL). Significance tested by one-way ANOVA.
